# Photodynamic therapy combined with antifungal drugs against chromoblastomycosis and the effect of ALA-PDT on *Fonsecaea in vitro*

**DOI:** 10.1371/journal.pntd.0007849

**Published:** 2019-10-31

**Authors:** Yongxuan Hu, Xinyu Qi, Hengbiao Sun, Yan Lu, Yanqing Hu, Xuyang Chen, Kangxing Liu, Yemei Yang, Zuhao Mao, Zhong Wu, Xianyi Zhou

**Affiliations:** 1 Department of Dermatology and Venerology, The 3rd Affiliated Hospital of Southern Medical University, Guangzhou, Guangdong, China; 2 Department of Clinical Laboratory, The 3rd Affiliated Hospital of Southern Medical University, Guangzhou, Guangdong, China; University of Tennessee, UNITED STATES

## Abstract

**Background:**

Chromoblastomycosis is a chronic skin and subcutaneous fungal infection caused by dematiaceous fungi and is associated with low cure and high relapse rates. In southern China, *Fonsecaea monophora* and *Fonsecaea pedrosoi* are the main causative agents.

**Principal findings:**

We treated 5 refractory and complex cases of chromoblastomycosis with 5-aminolevulinic acid photodynamic therapy (ALA-PDT) combined with oral antifungal drugs. The lesions improved after 4 to 9 sessions of ALA-PDT treatment at an interval of one or two weeks, and in some cases, mycological testing results became negative. The isolates were assayed for susceptibility to antifungal drugs and ALA-PDT *in vitro*, revealing sensitivity to terbinafine, itraconazole and voriconazole, with ALA-PDT altering the cell wall and increasing reactive oxygen species production.

**Conclusions:**

These results provide the basis for the development of a new therapeutic approach, and ALA-PDT combined with oral antifungal drugs constitutes a promising alternative method for the treatment of refractory and complex cases of chromoblastomycosis.

## Author summary

Chromoblastomycosis, a neglected tropical disease, is one of the most frequently encountered subcutaneous mycoses. The disease is usually caused by traumatic inoculation of a specific group of dematiaceous fungi. Chromoblastomycosisis characterized by slowly expanding skin lesions and is associated with low cure and high relapse rates. In recent years, effective methods, such as photodynamic therapy (PDT), have been employed for inhibiting the pathogen’s activity. The authors treated 5 refractory and complex cases of chromoblastomycosis with 5-aminolevulinic acid photodynamic therapy (ALA-PDT) combined with oral antifungal drugs. The lesions improved after 4 to 9 sessions of ALA-PDT treatment at an interval of one or two weeks, and in some cases, mycological testing results became negative. The authors also found that ALA-PDT alter the fungi cell wall and increase reactive oxygen species production. This research provides the basis for the development of a new therapeutic approach, and ALA-PDT combined with oral antifungal drugs constitutes a promising alternative method for the treatment of refractory and complex cases of chromoblastomycosis.

## Introduction

Chromoblastomycosis, a neglected tropical disease, is one of the most frequently encountered subcutaneous mycoses in tropical and subtropical regions. The disease is characterized by slowly expanding skin lesions and is associated with low cure and high relapse rates. Chromoblastomycosis is usually caused by traumatic inoculation of a specific group of dematiaceous fungi [1, 2]. To date, several species of pathogens have been reported to be involved in the disease etiology, including *Cladophialophora carrionii*, *Fonsecaea monophora*, *Fonsecaea pedrosoi*, *Fonsecaea nubica* and *Rhinocladiella aquaspersa* [1, 3]. In southern China, *F*. *monophora* and *F*. *pedrosoi* are the most common causal agents [4], with *F*. *nubica* and *Phialophora aquaspersa* being less common [5, 6].

Treatment of chromoblastomycosis remains a challenge due to its recalcitrant nature [7]. Indeed, some strains of *F*. *pedrosoi* are resistant to many antifungal drugs, and infection can be extremely difficult to eradicate [8, 9]. In general, treatment of cases caused by *F*. *monophora* is relatively easier than for cases caused by *F*. *pedrosoi* [10, 11]. In recent years, effective methods, such as photodynamic therapy (PDT), have been employed for inhibiting the pathogen’s activity. PDT is a minimally invasive approach in which photosensitizers are activated by exposure to low-intensity harmless visible light. Activation of photosensitizer results in the production of reactive oxygen species (ROS) and other reactive molecules, leading to damage at the site of infection and apoptosis in target cells. PDT has been applied to combat cancerous lesions, as well as some infectious diseases, especially human papillomavirus (HPV) infection, and a wide range of microorganisms has been demonstrated to be susceptible to antimicrobial PDT [12, 13]. This treatment may be considered an alternative for the management of some refractory and complex fungal infections [14], and antifungal PDT has been successfully employed against *Candida* species [15], dermatophytes [16], *A*. *fumigatus* [17] and *F*. *monophora* [3].

In this study, the clinical effect of 5-aminolevulinic acid (ALA)-PDT on chromoblastomycosis and its antifungal activity were evaluated *in vitro*. We describe 5 refractory and complex cases treated with ALA-PDT in combination with oral antifungal drugs. We observed a positive clinical effect, highlighting the efficiency of ALA-PDT against chromoblastomycosis. Considering that the majority of the research published to date has focused on *in vitro* trials, our clinical data can be considered a relevant source of information regarding antifungal ALA-PDT.

### Case 1

**Fig 1:**
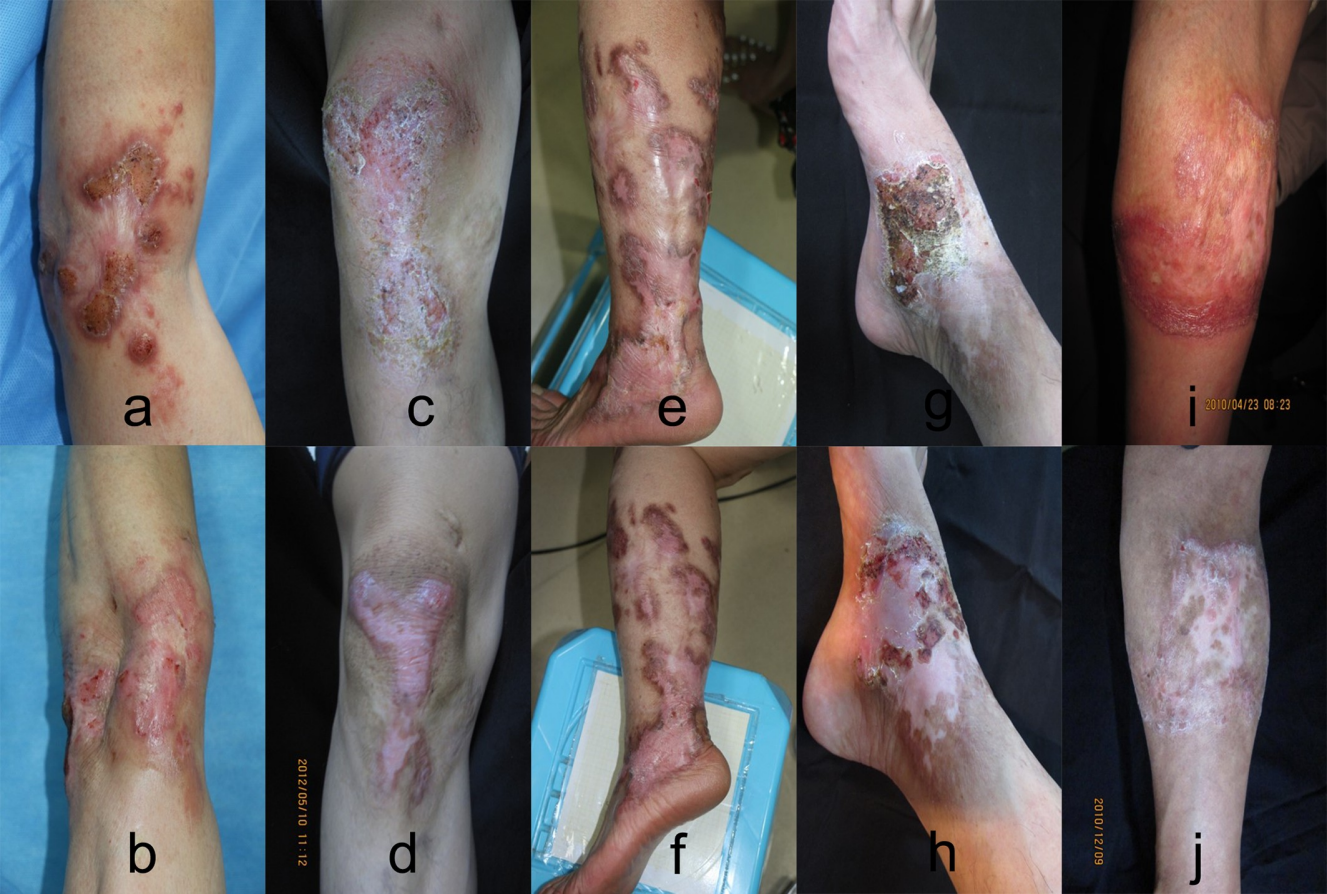
Clinical aspect of chromoblastomycosis lesions of patients before and after therapy. The clinical aspect of chromoblastomycosis lesions of patients (a, c, e, g, i) improved after ALA-PDT irradiation combined with antifungal drugs (b, d, f, h, j).

**Fig 2:**
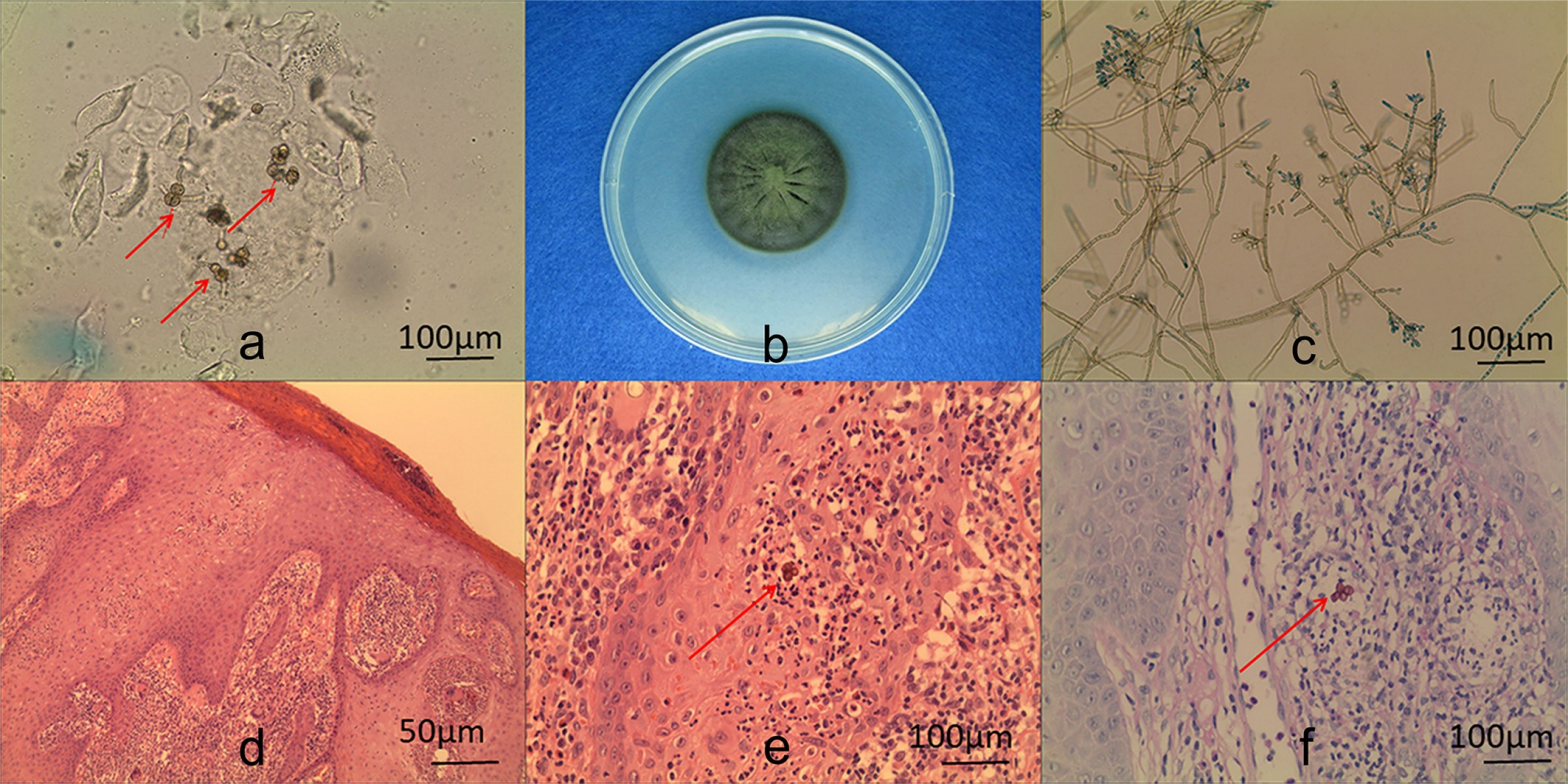
KOH examination of scales, histopathological examination of biopsies, and culture of isolates obtained from patient. KOH wet mount of the sample from a lesion, showing muriform cells (×400) (a). Macroscopic appearance of a *Fonsecaea* colony (b). Microculture of *F*. *nubica* (×400) (c). Muriform cells on histopathological examination of a biopsy (hematoxylin-eosin stain) (d: ×100; e: ×400). Periodic acid-Schiff (PAS) stain revealed muriform cells (f).

A 50-year-old male farmer residing in Guangzhou Panyu, China, presented to our outpatient clinic on March 29, 2018. He complained of an itchy erythematous plaque surrounded with veracious hyperplasia on the left elbow (Fig 1a). The lesion started 10 years ago after local trauma and enlarged gradually. The patient had visited other hospitals, and a clinical diagnosis of deep mycosis (without isolation of pathogens) was made. Before he visited our hospital, itraconazole and terbinafine had been used for more than 1 year, but the lesion did not improve. At our hospital, both examination of potassium hydroxide mounts (Fig 2a) and histopathology of the lesion revealed dematiaceous muriform cells (Fig 2d, 2e and 2f), supporting a diagnosis of chromoblastomycosis. Based on a mycological analysis and DNA sequencing, the etiological agent was identified as *F*. *nubica*. His family history and past medical history were unremarkable. Underlying diseases or immunocompromised conditions were not present in this patient. For treatment, ALA-PDT (concentration of 20%, duration time of 4 h) irradiation was combined with oral itraconazole 200 mg/day. He received this therapy 4 times from April 4, 2018, to May 4, 2018, at an interval of 1 week. The lesions were obviously improved clinically (Fig 1b), but fungal testing was still positive. After ALA-PDT treatment cessation, oral itraconazole 200 mg/day alone was administered for 1 year (Table 1). No new lesions developed, but the plaque did not disappear. The patient remains under follow-up (Table 2).

**Table 1:** MICs and FICIs of ITZ/TBF and VOR/TBF against isolates obtained from patients.

The isolation	Drugs	MIC (μg/mL)			MICs of the combination			FICI
		ITZ	VOR	TBF	ITZ	VOR	TBF	
*F*. *nubica* (from case 1)	ITZ/TBF	1	--	0.25	0.25	--	0.125	0.75
	VOR/TBF	--	0.25	0.25	--	0.0313	0.125	0.625
*F*. *pedrosoi* (from case 2)	ITZ/TBF	1	--	0.125	0.5	--	0.0625	1
	VOR/TBF	--	0.25	0.125	--	0.0625	0.0625	0.75
*F*. *pedrosoi* (from case 3)	ITZ/TBF	1	--	0.125	0.25	--	0.125	1.25
	VOR/TBF	--	0.125	0.125	--	0.0625	0.125	1.5
*F*. *monophora* (from case 4)	ITZ/TBF	1	--	0.125	0.25	--	0.0625	0.75
	VOR/TBF	--	0. 5	0.125	--	0.125	0.0625	0.75
*F*. *monophora* (from case 5)	ITZ/TBF	1	--	0.125	0.25	--	0.0625	0.75
	VOR/TBF	--	0. 0625	0.125	--	0.0313	0.0625	1

MIC, minimal inhibitory concentration; FICI, fractional inhibitory concentration index; ITZ, itraconazole; TBF, terbinafine; VOR, voriconazole

**Table 2:** Photodynamic therapy combined with antifungal drugs for 5 cases of chromoblastomycosis.

case	Type of infections	Strain	ITS analyses (GenBank accession number)	Antifungal	Antifungal dose, administration	PS	Light source	Wavelength (nm)	Power (mW cm^−2^)	Fluence (J cm^−2^) and aPDT sessions	Observed effect
Case 1	left elbow	*F*. *nubica*	MK931433	Itraconazole	400 mg day^−1^ oral	5- ALA	LED	635	36.8	4 sessions weekly	The lesions were obviously improved, but the plaque did not disappear. Fungal testing was positive.
Case 2	right knee	*F*. *pedrosoi*	MK959027	Terbinafine	250 mg day^−1^ oral	5- ALA	LED	635	36.8	4 sessions weekly	The plaque disappeared, with some hypopigmentation remaining. Fungal testing was negative.
Case 3	left leg	*F*. *pedrosoi*	MK959028	Itraconazole and Terbinafine	400 mg and 250 mg day^−1^ oral	5- ALA	LED	635	36.8	4 sessions weekly	The lesions were partial improved, but the plaque and nodules did not disappear. Fungal testing was positive.
Case 4	right ankle	*F*. *monophora*	JN629042	Terbinafine	250 mg day^−1^ oral	5- ALA	LED	635	36.8	2 (9 sessions weekly)	Lesions improved clinically, with no recurrence.
Case 5	right arm	*F*. *monophora*	JN629041	Terbinafine	250 mg day^−1^ oral	5- ALA	LED	635	36.8	5 sessions weekly (2 periods)	Lesions improved clinically, with no mycological or complete clinical cure.

5-ALA: 5-aminolevulinic acid; aPDT: antimicrobial photodynamic therapy; LED: light-emitting diode; PS: photosensitizer.

### Case 2

A 53-year-old male sailor residing in Guangzhou, China, presented to our outpatient clinic on April 7, 2011. He complained of an itchy erythematous plaque surrounded with veracious hyperplasia on the right knee (Fig 1c). The lesion started 15 years ago after local trauma and enlarged gradually. The patient had visited other hospitals many times, and a clinical diagnosis of eczema or neurodermatitis was made. Before half-one year, he received a clinical diagnosis of deep mycosis at another hospital, and fluconazole and itraconazole had been employed for more than 6 months. At our hospital, both direct examination of potassium hydroxide mounts and histopathology revealed dematiaceous muriform cells, which supported a diagnosis of chromoblastomycosis. The etiological agent was identified as *F*. *pedrosoi* based on mycological and DNA sequencing analyses. His family history and past medical history were unremarkable; underlying diseases and immunocompromised conditions were not present in this patient. As treatment, ALA-PDT (concentration of 20%, duration time of 4 h) irradiation was combined with oral terbinafine 250 mg/day from April 21, 2011, to May 21, 2011, for a total of 4 times and at an interval of 1–2 weeks. The lesions were notably improved clinically, and fungal examination testing was negative. After ALA-PDT treatment cessation, oral terbinafine 250 mg/day alone was administered for six months (Table 1). No new lesions developed. At one year later, the plaque had disappeared, leaving only some hypopigmentation (Fig 1d) (Table 2).

### Case 3

A 50-year-old female farmer residing in Shaoguan, China, presented to our outpatient clinic on May 10, 2012 complaining of an itchy erythematous plaque and nodules on the left leg (Fig 1e). The lesion began 25 years ago after local trauma and enlarged gradually; 10 years ago, the patient was diagnosed with deep mycosis at another hospital. She had received different treatments, such as itraconazole 200 mg/day for more than 5 years and/or terbinafine 250 mg/day for 2 years, with partial improvement. At our hospital, both direct examination of potassium hydroxide mounts and histopathology showed dematiaceous muriform cells, and the diagnosis of chromoblastomycosis was made. Mycological and DNA sequencing analyses identified the etiological agent as *F*. *pedrosoi*. She had no relevant family history or past medical history, with no underlying diseases or immunocompromised conditions. ALA-PDT (concentration of 20%, duration time of 4 h) irradiation of the left ankle combined with itraconazole 200 mg/day and terbinafine 250 mg/day orally from July 2, 2013, to July 30, 2013, at an interval of 1–2 weeks for a total of 3 times, was adopted as treatment. Although the lesions partially improved clinically, fungal testing was positive. After cessation of ALA-PDT treatment, oral itraconazole 200 mg/day and terbinafine 250 mg/day were administered for more than 2 years (Table 1), though the plaque and nodules did not disappear (Fig 1f). The patient is still under follow-up (Table 2).

### Case 4

A 50-year-old male farmer had complained of an itchy erythematous plaque surrounded with veracious hyperplasia on his right ankle (Fig 1g) [18]. The lesion started 30 years ago after local trauma and grew over time. The patient had visited another hospital, receiving a clinical diagnosis of deep mycosis (without pathogen isolation). Before he visited our hospital, he had been using itraconazole and fluconazole for more than 5 years. Potassium hydroxide mount and histopathology examinations at our hospital supported the diagnosis of chromoblastomycosis, and the isolate was identified as *F*. *monophora* by mycological analysis and DNA sequencing. ALA-PDT was administered 18 times and combined with oral terbinafine 250 mg/day (Table 1). The lesions were markedly improved clinically, with negative fungal tests. The plaque disappeared, leaving some hypopigmentation (Fig 1h) (Table 2).

### Case 5

A 55-year-old male farmer had an itchy erythematous plaque surrounded with verrucous hyperplasia on the medial side of his right arm (Fig 1i) [3]. The lesion appeared after a local trauma on this arm 13 years ago and gradually became larger. He had visited other hospitals; a diagnosis of deep mycosis was made, but he only received external antifungal treatment. Chromoblastomycosis was diagnosis after examination of potassium hydroxide mounts and histopathology. Mycological and DNA sequencing analyses showed the isolate to be *F*. *monophora*. As treatment, ALA-PDT was administered 10 times and combined with terbinafine 250 mg/day orally (Table 1). Despite obvious clinical improvement in the lesions, positive fungal tests were obtained (Fig 1j). Nonetheless, mycological testing was negative six months later. The patient remains under follow-up (Table 2).

## Materials and methods

### Ethics statement

The study was approved by the Ethics Committee of the Third Affiliated Hospital of Southern Medical University. All subjects were adults and provided written informed consent.

### Identification of isolate

KOH examination of scales from lesions and histopathology of biopsy specimens were performed. Macroscopic and microculture of the isolates ensued [3, 10, 18].

DNA was extracted using 6% InStaGene Matrix (BioRad, Hercules, CA, USA). Ribosomal DNA ITS regions were amplified using a Biometra T-Gradient Thermoblock (Whatman Biometra, Goettingen, Germany) with primers ITS-5 (5’-GGAAGTAAAAGTCGTAACAAGG-3’) and ITS-4 (5’-TCCTCCGCTTATTGATATGC-3’). PCR was carried out at 94°C for 5 min, followed by 30 cycles at 94°C for 60 s, annealing at 55°C for 90 s and an extension at 72°C for 90 s; the reaction continued at 72°C for 10 min. The DNA fragments were sequenced using an ABI PRISM 3100 sequencer (Applied Biosystems, Foster City, CA, USA).

### Antifungal susceptibility testing

The clinical isolate was subjected to antifungal susceptibility testing according to CLSI guidelines (M38-A document), as previously described [3, 18], and the MICs of antifungal combinations were determined according to previously described methods [3, 18]. Itraconazole (Xian-Janssen Pharmaceutical Ltd. Xi’ an, China), terbinafine (Beijing Novartis Pharmaceutical Ltd. (Beijing, China)) and voriconazole (Sigma, USA) were dissolved in 100% DMSO as a stock solution (3200 μg/mL). Drugs were diluted to obtain final concentrations, with itraconazole and voriconazole from 0.008 to 8 μg/mL and terbinafine from 0.008 to 0.5 μg/mL. Isolates were sub-cultured, and spores from colonies were collected and adjusted with saline to achieve an inoculum concentration of 10^6^ conidia/mL. Each suspension was diluted 1:50–100 with RPMI 1640 to obtain the final test inoculum (0.4–5×10^4^ conidia/mL). Conidial suspension of each of the tested strains were cultivated on RPMI 1640 medium for 7 days at 35°C. *Candida parapsilosis* ATCC22019 (CBS604), obtained from Centraalbureau voor Schimmelcultures (CBS, the Netherlands), was used as a quality control. The final test inoculum concentration was 0.5–2.5×10^3^ conidia/mL.

### Antifungal effect of ALA-PDT/Itraconazole on *F*. *monophora* by transmission electron microscopy

Isolate of *F*. *monophora* from the patient 4 was used in this experiment. Fungus culture and ALA-PDT were performed as previously described [3,18]. The first culture of *F*. *monophora* (master plates) was carried out on Kimmig-Agar-Plates (Merck, Darmstadt, Germany) for 3–4 weeks at room temperature in the dark. Liquid cultures of *F*. *monophora* were prepared by inoculation of Sabouraud glucose (2%) broth (Heipha Diagnostika, Heidelberg, Germany) with fungi from master plates. Liquid cultures were continuously shaken at 50 rpm on a shaker (Promax 2020; Heidolph, Schwabach, Germany) to achieve dynamic access of ALA to the fungi growth.

ALA was kindly provided by Schering AG (Berlin, Germany). Two stock solutions of ALA at concentrations of 3.33 M (stock solution I) and 33.3 M (stock solution II) were prepared. Prior to use, both solutions were filter-sterilized (0.2 μm, Schleicher & Schuell, Dassel, Germany) and added to growth media at the appropriate concentration (final concentration was 10mM). The antifungal drug Itraconazole was provided by Sigma, and the final concentration was 1μg/mL.

A Zeiss KL 2500 LED at a wavelength of 635 nm was used for all irradiation experiments. The fluency rate of the illuminator was 36.8 mW/cm^2^. White light was applied (unfiltered). To minimize the non-uniformity of the light output across the irradiated area, the distance from the fiber optic to the surface of the plates was 5 cm. Plates containing *F*. *monophora* were exposed to a treatment equivalent of 10 J. After 20 minutes of irradiation, plates containing *F*. *monophora* were fixed and then observed by transmission electron microscopy. All tests were performed in triplicate.

### Antifungal effect of ALA-PDT/Itraconazole on *F*. *monophora in vitro* by ROS

Isolate of *F*. *monophora* from the patient 4 was used. The fungal culture, ALA-PDT and antifungal drug Itraconazole were performed as described above. Suspensions were centrifuged, and the supernatant was discarded; the fungal pellet was resuspended in 10 μM DCFH-DA probe solution and incubated for 30 minutes at 37°C. The fluorescence intensity of ROS was measured by flow cytometry after washing with PBS and resuspending the fungi. All tests were performed in triplicate.

### Plate counts to quantify the number of dead verses live cells

Isolate of *F*. *monophora* from the patient 4 was used. Fungus culture and plate counts were performed as previously described [18]. Plates with *F*. *monophora* were prepared with ALA (10mM) and/or Itraconazole (1μg/mL), then exposed to a treatment equivalent to 10 J. After 7 days of further incubation at room temperature (25°C), plates of *F*. *monophora* were evaluated for killing effects of ALA-PDT and/or Itraconazole, and CFU counting was made. All tests were made in triplicate.

## Results

### Identification of isolate

KOH wet mounts of the sample from the lesion of Case 1 showed muriform cells (dark-brown large cells) (Fig 2a). Histopathology of a biopsy specimen revealed mild acanthosis of the epidermis and granulomatous inflammation around the entire dermis. Muriform cells were observed in micro-abscess or giant cells (Fig 2d and 2e). Periodic acid-Schiff (PAS) staining revealed muriform cells (Fig 2f).

### DNA sequence analyses

The DNA sequence of the isolate from Case 1 showed 100% homology with the type strain NYSM-0270 in GenBank (KY432481.1) and was confirmed as *F*. *nubica*. The sequence data were deposited in GenBank under accession number MK931433.

### Antifungal susceptibility testing

The clinical isolates were subjected to antifungal susceptibility testing, and the results are shown in Table 1. Itraconazole, voriconazole and terbinafine all demonstrated considerable efficacy against the clinical isolate from Case 1. The minimal inhibitory concentrations (MICs) were 1, 0.25 and 0.25 μg/mL for itraconazole, voriconazole and terbinafine, respectively, and the fractional inhibitory concentration index (FICI) was 0.75 and 0.625, respectively.

### Antifungal effect of ALA-PDT/Itraconazole on *F*. *monophora* by transmission electron microscopy

**Fig 3:**
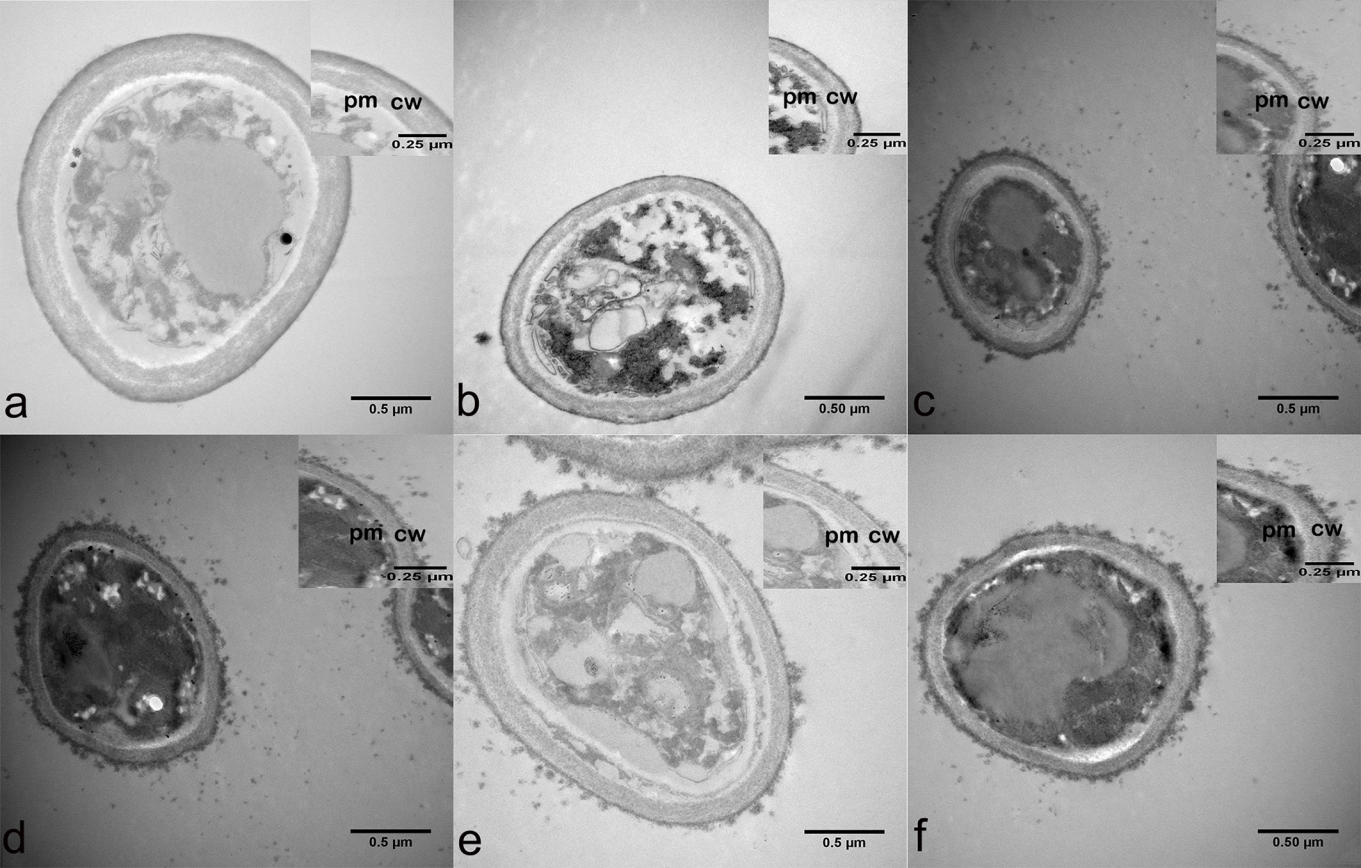
Antifungal effect of ALA-PDT/Itraconazole on *F*. *monophora* by transmission electron microscopy. Before ALA-PDT and/or Itraconazole treatment, the fungal spores presented a round-shape morphology with a homogenous cytoplasm, linear plasma membrane (pm) and a cell wall (cw) with two distinct layers: an inner electron dense and outer fibrillar layer. (a) 0 M ALA, and without light irradiation; (b) 10 M ALA, and without light irradiation; (c) 0 M ALA, and with light irradiation; (d) 10 M ALA, and with light irradiation; (e) 1μg/mL Itraconazole; (f) 1μg/mL Itraconazole, and 10 M ALA with light irradiation.

Transmission electron microscopy employed to evaluate the photo-killing effect of ALA-PDT (Fig 3). Before ALA-PDT treatment, the fungal spores presented a round-shape morphology with a homogenous cytoplasm, linear plasma membrane and a cell wall with two distinct layers: an inner electron dense and outer fibrillar layer. After ALA-PDT treatment, the spores exhibited an increase of electron-lucent vacuoles as well as detachment of the outermost fibrillar layer.

### Antifungal effect of ALA-PDT/Itraconazole on *F*. *monophora* by ROS

**Fig 4:**
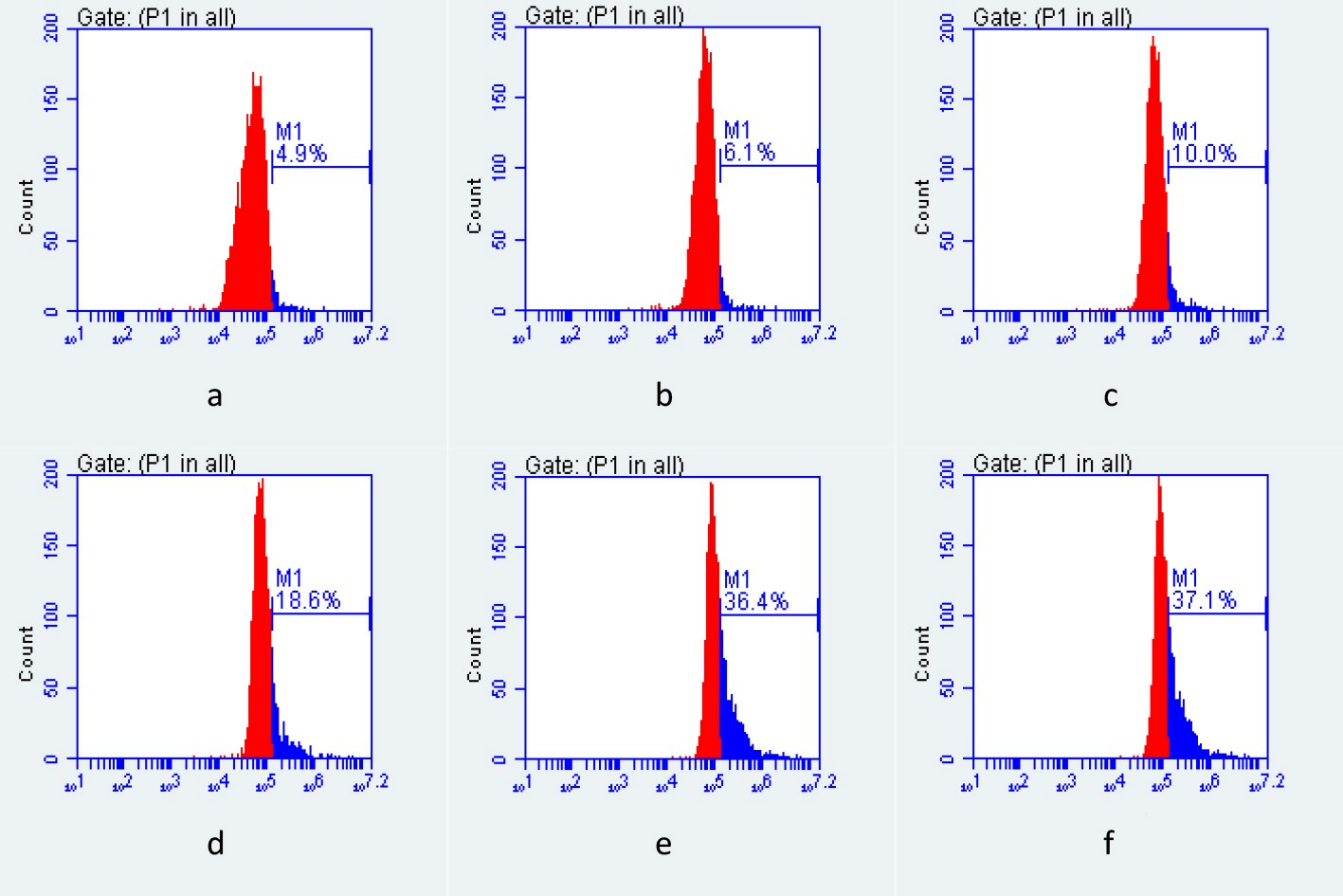
Antifungal effect of ALA-PDT/Itraconazole on *F*. *monophora* by ROS. The fluorescence intensity of ROS was measured by flow cytometry, and ROS increased significantly after ALA-PDT and/or Itraconazole. (a) 0 M ALA, and without light irradiation; (b) 10 M ALA, and without light irradiation; (c) 0 M ALA, and with light irradiation; (d) 10 M ALA, and with light irradiation; (e) 1μg/mL Itraconazole; (f) 1μg/mL Itraconazole, and 10 M ALA with light irradiation.

The fluorescence intensity of ROS was measured by flow cytometry, as shown in Fig 4. In this experiment, we clearly demonstrated that ROS production increased after ALA-PDT and Itraconazole.

### Plate counts to quantify the number of dead verses live cells

**Fig 5:**
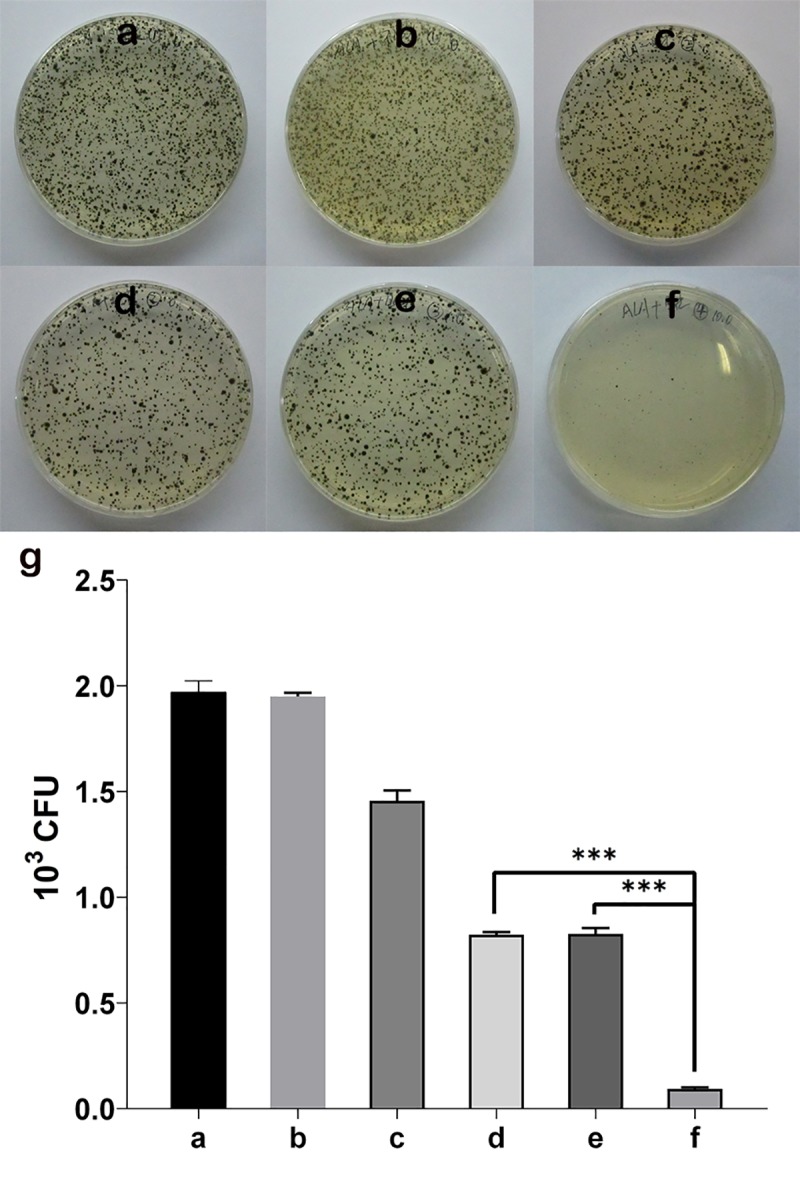
Plate counts to quantify the number of dead verses live cells. Plates of *F*. *monophora* were evaluated for killing effects of ALA-PDT and/or Itraconazole, and CFU counting was made. (a) 0 M ALA, and without light irradiation; (b) 10 M ALA, and without light irradiation; (c) 0 M ALA, and with light irradiation; (d) 10 M ALA, and with light irradiation; (e) 1μg/mL Itraconazole; (f) 1μg/mL Itraconazole, and 10 M ALA with light irradiation; (g) the statistics of the antifungal effect of ALA-PDT and/or Itraconazole in *F*. *monophora* (***P<0.01).

After 7 days of further incubation at 25°C, plates of *F*. *monophora* were evaluated for killing effect of ALA-PDT and/or antifungus by CFU quantification (Fig 5). In this experiment, we could clearly demonstrate that the growth-inhibiting effect of ALA-PDT and/or Itraconazole.

## Discussion

The cases of chromoblastomycosis discussed herein were caused by *F*. *nubica*, *F*. *pedrosoi* and *F*. *monophora*. *F*. *monophora*, one of the most common causes of the mycosis in southern China, was first identified by De Hoog [19]. Among various antifungal drugs, itraconazole and terbinafine are considered to be effective, either by drug sensitivity testing *in vitro* or clinical application. The patients in this study were initially treated with itraconazole and/or terbinafine or their combination, with only partial improvement or without a good response. However, when ALA-PDT was employed, the lesions improved partially or significantly.

Topical PDT can cause destruction of selected cells via the combination of a photosensitizer and visible light, as first employed in the oncological field [12]. Currently, PDT is a well-established treatment for a variety of malignant skin tumors, including non-melanoma skin cancer, actinic keratosis, and some inflammatory diseases, such as acne vulgaris, photorejuvenation and hidradenitis suppurativa [13]. Moreover, PDT treatment, alone or in combination, has been extended to antimicrobial chemotherapy. PDT for treatment of infections caused by *Candida* species [15], dermatophytes [16], *Aspergillus* [17] and *Fonsecaea* [3, 18] has been reported with promising results, with related investigations both *in vitro* and *in vivo* [3, 18]. Although complete healing clinically and mycologically was not achieved in these cases, the lesions were improved greatly or partially. Our previous *in vitro* study showed that the growth-inhibiting effect of ALA-PDT on *F*. *monophora* is compatible with a clinical response [18].

In the present study, isolates of *Fonsecaea* were assessed for antifungal susceptibility, and all five isolates showed good sensitivity to terbinafine, itraconazole and voriconazole. This also suggested that determination of *in vitro* susceptibility profiles may be useful for identifying intrinsic microbiological resistance to antifungal drugs but does not predict clinical response [20]. The patient’s status, drug combination and longer courses of drug administration may also be important factors for the treatment effect. Our previous *in vivo* studies revealed a synergistic effect of terbinafine and itraconazole on clinical isolates of *F*. *monophora* [3, 10, 20].

Although chromoblastomycosis is associated with low cure and high relapse rates [2], many cases caused by *F*. *monophora* have been successfully treated. It is generally believed that *F*. *monophora* displays a better therapeutic effect than does *F*. *pedrosoi* [9, 10]. Here, we describe five refractory cases of chromoblastomycosis: one caused by *F*. *nubica*, two caused by *F*. *monophora*, and another two caused by *F*. *pedrosoi*. It is possible that fibrosis is a key impediment to access of an antimycotic. In general, itraconazole and/or terbinafine combined with PDT are promising methods for treating refractory chromoblastomycosis cases. It has been reported that miconazole can be used to increase the efficacy of PDT against *C*. *albicans* and that its mechanism of action is likely to be multifactorial [21]. Itraconazole and/or terbinafine may also increase the efficacy of PDT against *Fonsecaea*, but this should be confirmed *in vitro*.

*In vitro* PDT against *F*. *pedrosoi* and *Cladophialophora carrionii* has been reported [22]. In our previous study, *F*. *monophora* was treated with ALA and irradiated to achieve photodynamic inactivation of this fungus, and we clearly demonstrated the growth-inhibiting effect of ALA-PDT [18]. Effects on biofilm formation and cell wall structure destruction were considered to be the mechanism of action [23, 24, 25]. This study was performed to investigate the mechanism, as to whether the cell wall of *F*. *monophora*, *F*. *pedrosoi* or *F*. *nubica* can be changed by ALA-PDT *in vitro*.

The ROS pathway is considered to be a mechanism of cell death, and *in vitro* studies have shown that ALA-PDT inactivates *F*. *monophora* by directly killing conidia via ROS-dependent oxidative damage [26]. Direct determination of ROS in fungi by fluorescence showed that ROS levels were significantly increased in the isolates of *F*. *monophora*, *F*. *pedrosoi* and *F*. *nubica* from the patients after ALA-PDT.

In CFU quantification experiment of Fig 5, we could clearly demonstrate that the growth-inhibiting effect of ALA-PDT and/or Itraconazole, the results showed that there was a synergistic effect of itraconazole + ALA-PDT. But it was unclear from the TEM as there was as strong of an effect from light alone, from drug or chemical and light combinations. Also, for the ROS production in Fig 4 there was no different between drug and ALA-PDT and ALA-PDT alone. The reason for this may be itraconazole induce changes in the cell membrane and change the thickness of cell wall of fungi [25, 27], ALA-PDT can inhibit fungi by producing a large number of ROS [28, 29]. Of course, in addition to these pathways, antifungal drugs and ALA-PDT may have other antifungal mechanisms. Because of these different antifungal mechanisms, it can achieve better synergistic effect of itraconazole and ALA-PDT.

To enhance uptake and conversion of ALA by *Fonsecaea*, ALA should be esterified. Further experiments should be performed to substantiate the growth-limiting properties of ALA-PDT to obtain a successful treatment of chromoblastomycosis infection and elucidate the mechanism by which ALA-PDT synergizes or enhances antifungal drug sensitivity *in vitro* and *in vivo*.
